# Efficacy of Shenglin decoction in preventing acute severe lymphocytopenia in patients with non-small cell lung cancer undergoing concurrent chemoradiotherapy: a study protocol for a randomized controlled trial

**DOI:** 10.3389/fonc.2024.1378662

**Published:** 2024-05-08

**Authors:** Jiayao Deng, Cuicui Gong, Qi Xiao, Bo Xu, Huakang Li, Ziliang Wu, Qian Xiao, Pengxuan Gu, Qiang Li, Bin Li, Yue Wang, Bing Lin, Ke Xu

**Affiliations:** ^1^ Department of Oncology, Hospital of Chengdu University of Traditional Chinese Medicine, Chengdu, China; ^2^ Clinical School, Chengdu University of Traditional Chinese Medicine, Chengdu, China; ^3^ Health Management Center, Hospital of Chengdu University of Traditional Chinese Medicine, Chengdu, China; ^4^ Department of Radiation Oncology, Radiation Oncology Key Laboratory of Sichuan Province, Sichuan Clinical Research Center for Cancer, Sichuan Cancer Hospital and Institute, Sichuan Cancer Center, Affiliated Cancer Hospital of University of Electronic Science and Technology of China, Chengdu, China

**Keywords:** acute severe lymphopenia, traditional Chinese medicine, clinical trial protocol, non-small cell lung cancer, concurrent chemoradiotherapy

## Abstract

**Background:**

Definitive concurrent chemoradiotherapy (CCRT) followed by maintenance therapy with immune checkpoint inhibitors offers the best chance of cure for patients with stage III non-small cell lung cancer (NSCLC). A significant challenge in this regimen is the occurrence of acute severe lymphopenia (ASL), which can compromise treatment efficacy. Currently, there are no effective strategies for preventing and treating ASL. Shenglin decoction (SLD), a traditional Chinese herbal medicine formulation, has demonstrated preliminary efficacy in mitigating ASL. However, robust evidence from clinical trials and a clear understanding of its mechanism of action are still needed. This study aims to comprehensively assess the efficacy, safety, and underlying mechanisms of SLD in the prevention of ASL.

**Methods:**

This prospective, dual-center, open-label, randomized controlled trial will enroll 140 stage III NSCLC patients. Participants will be randomly allocated in a 1:1 ratio to a control group or an experimental group. Both groups will undergo definitive CCRT. Alongside the commencement of CCRT, the experimental group will receive an additional oral SLD intervention for a duration of three months. The primary outcome is the incidence rate of ASL, defined as the proportion of patients who experience at least one instance of a total lymphocyte count falling below 0.5 × 10^9 cells/L within 3 months of initiating CCRT treatment. Additionally, 16S rRNA gene sequencing analysis of fecal samples to assess gut microbiota, as well as metabolomic analysis of fecal/blood samples, will be conducted to explore potential mechanisms.

**Discussion:**

This study protocol aims to rigorously evaluate the efficacy and safety of SLD, as well as elucidate its mechanism of action in preventing ASL. Successful outcomes could establish SLD as an evidence-based intervention for ASL prevention in NSCLC patients undergoing CCRT.

**Trial Registration:**

The trial was registered at the Chinese Clinical Trials Registry (ChiCTR2300071788, https://www.chictr.org.cn/).

## 1 Introduction

In the current era of immunotherapy, maintenance treatment with immune checkpoint inhibitors (ICI) following definitive concurrent chemoradiotherapy (CCRT) has become the new standard of care for patients with stage III non-small cell lung cancer (NSCLC) (1). A prevalent challenge in this context is the occurrence of acute severe lymphopenia (ASL), defined as a total lymphocyte count (TLC) less than 0.5 × 10^9 cells/L within three months post-CCRT initiation (2), and classified as a grade 3-4 adverse event as per the Common Terminology Criteria for Adverse Events (CTCAE). Two large retrospective studies have shown that the incidence of ASL in stage III NSCLC patients following CCRT reached 92% (330/362) (3) and 88% (532/604) (4) respectively. In the era when CCRT alone was considered the standard treatment for stage III NSCLC patients, it was already observed that an association exists between ASL and poorer survival rates (5). Recent studies further suggest that ASL impairs the efficacy of subsequent ICI maintenance therapy in this population (6). Given the negative impact of ASL on the effectiveness of both CCRT and ICI treatments, preventing lymphocyte depletion and promoting lymphocyte recovery is of paramount importance (7). However, most current pharmacological studies for the management of ASL are in the preclinical and early clinical trial stages, with no approved drugs for clinical use (8).

The Shenglin decoction (SLD), a proprietary Chinese herbal medicine formula developed by our research team, targets the management of ASL. This formulation comprises fifteen distinct herbal ingredients. **Table 1** delineates the exact composition of each individual dose of SLD. Every herb incorporated in the SLD formula is enumerated in the 2020 edition of the Pharmacopoeia of China, adhering to stringent standards of legality, safety, and efficacy. Our preliminary retrospective analysis, encompassing stage III NSCLC patients treated in two tertiary care hospitals from June 2021 to January 2023, has indicated SLD’s potential in ASL mitigation (the data have not yet been formally published). During this period, 89 patients used SLD prophylactically against ASL during CCRT. To control potential confounding factors, propensity score matching was utilized to select 89 patients as a control group, who did not receive any Chinese herbal treatment during CCRT in the same period. The analysis revealed ASL incidences of 86.5% in the control group versus 60.7% in the SLD group (*P* < 0.001). Encouraged by these results, we aim to conduct a randomized controlled clinical trial to further assess SLD’s efficacy and safety. Moreover, the exact mechanism of SLD’s action remains to be elucidated. We hypothesize that its effects might be mediated through alterations in gut microbiota and its metabolites. To investigate this, the proposed study will include 16S rRNA gene sequencing for gut microbiota analysis and metabolomic profiling of fecal/blood samples utilizing gas chromatography-time of flight mass spectrometry technology.

**Table 1:** Composition of Shenglin decoction.

Name of the Drug	Family name	Part & form used	Dosage(g)
Dang Gui [*Angelica sinensis* (Oliv.) Diels.]	Apiaceae	Root	4
Chuan Xiong [*Ligusticum chuanxiong* Hort.]	Apiaceae	Rhizome	3
Shu Di Huang [*Rehmannia glutinosa* Libosch.]	Orobanchaceae	Processed root	4
Bai Shao [*Paeonia lactiflora* Pall.]	Paeoniaceae	Root	5
Sheng Shai Shen [*Panax ginseng* C.A.Mey.]	Araliaceae	Root	4
Gan Cao [*Glycyrrhiza uralensis* Fisch.]	Fabaceae	Root	3
Fu Ling [*Poria cocos* (Schw.) Wolf.]	Polyporaceae	Sclerotium	4
Bai Zhu [*Atractylodes macrocephala* Koidz.]	Asteraceae	Rhizome	5
Huang Qi [*Astragalus membranaceus* (Fisch.) Bunge]	Fabaceae	Root	6
Jin Yin Hua [*Lonicera japonica* Thunb.]	Caprifoliaceae	Flower	5
Lian Qiao [*Forsythia suspensa* (Thunb.) Vahl]	Oleaceae	Fruit	4
Mai Dong [*Ophiopogon japonicus* (Thunb.) Ker Gawl.]	Asparagaceae	Tuber	4
Nan Sha Shen [*Adenophora stricta* Miq.]	Campanulaceae	Root	5
Dan Shen [*Salvia miltiorrhiza* Bunge]	Lamiaceae	Root	5
San Qi [*Panax notoginseng* (Burk.) F.H.Chen]	Araliaceae	Root	2

## 2 Materials and methods

### 2.1 Study design

**Figure 1:**
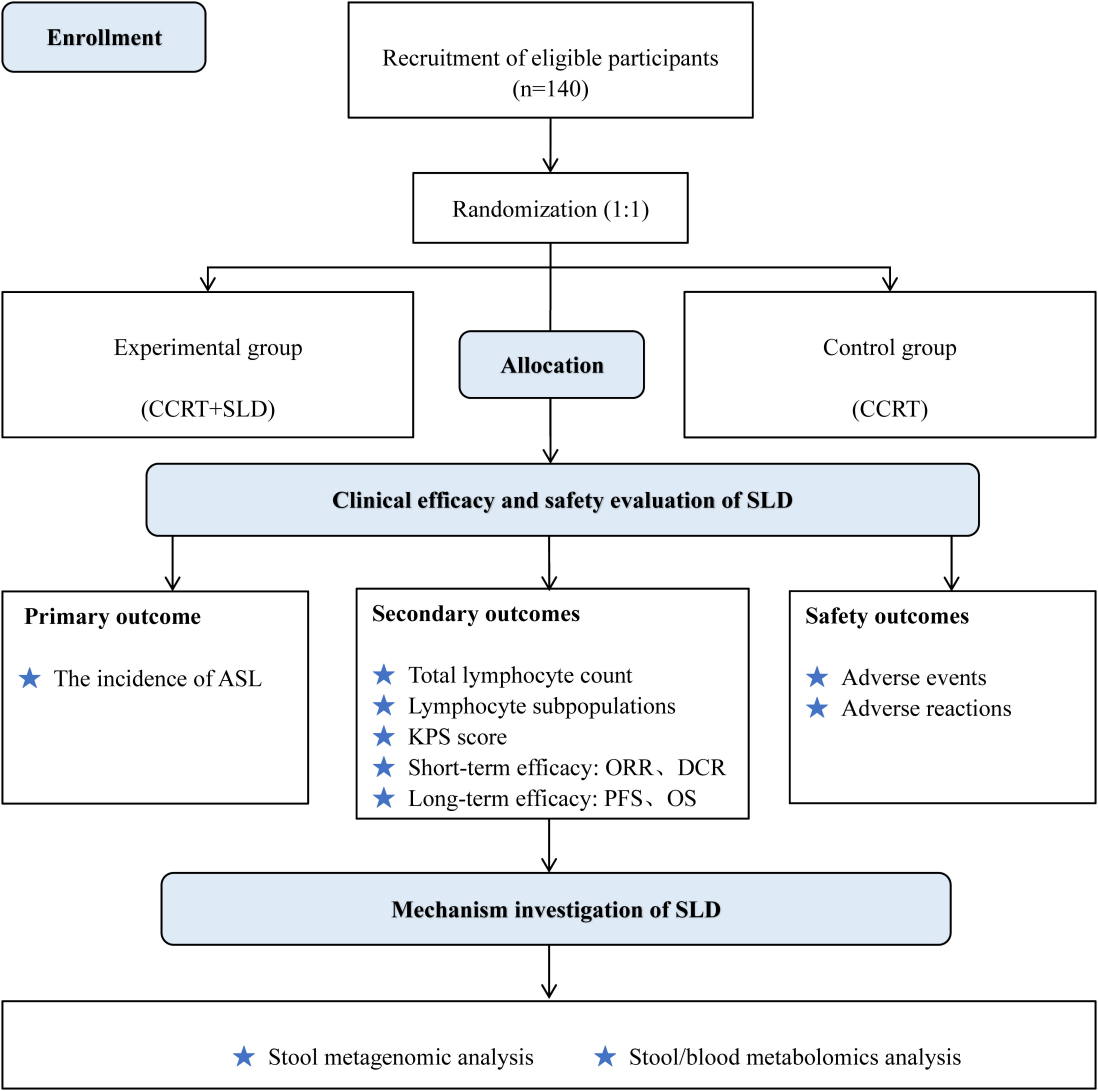
The flow diagram of this study. CCRT,concurrent chemoradiotherapy; ASL,acute severe lymphopenia; SLD,shenglin decoction; PFS,progression free survival; OS,overall survival; ORR, objective response rate; DCR, disease control rate.

This study is a prospective, dual-center, open-label, randomized controlled trial. It plans to recruit 140 patients with stage III NSCLC, who will be randomly assigned in a 1:1 ratio to either a control group (CCRT only) or an experimental group (CCRT + SLD). The primary outcome measure is the incidence rate of ASL. The study flow chart is shown in **Figure 1**.

### 2.2 Participants

#### 2.2.1 Recruitment

The study will recruit 140 suitable participants from stage III NSCLC patients receiving CCRT at Chengdu University of Traditional Chinese Medicine Affiliated Hospital and Sichuan Cancer Hospital. Participants will be selected based on predefined inclusion and exclusion criteria. Researchers will explain the study’s purpose, requirements, interventions, and all potential risks to potential patients, ensuring that they understand the study parameters before signing the informed consent form. Eligibility for the study will be jointly confirmed by a practicing oncologist and a practicing traditional Chinese medicine doctor.

#### 2.2.2 Inclusion criteria

(1) Age ranging from 18 to 75 years.
(2) Histopathologically confirmed NSCLC diagnosis.
(3) Stage III disease (according to the American Joint Committee on Cancer, 8th edition).
(4) Clinically assessed as inoperable and planned for CCRT.
(5) White blood cells, neutrophils, lymphocyte counts ≥ lower limit of normal (LLN) and ≤ upper limit of normal (ULN), hemoglobin ≥ 100 g/L, platelets ≥ 100 × 10^9/L, transaminases ≤ 1.5 ULN, bilirubin ≤ 1.0 ULN, creatinine ≤ 1.0 ULN, creatinine clearance rate ≥ 60 mL/min.
(6) Karnofsky Performance Status (KPS) score ≥ 70.

#### 2.2.3 Exclusion criteria

(1) Concurrent hematologic or immunologic diseases (such as leukemia, myelodysplastic syndromes, acquired immunodeficiency syndrome, etc.).
(2) Previous exposure to radiotherapy, chemotherapy, immunotherapy, or biological therapy.
(3) Known or suspected allergy or intolerance to any of the herbal medicines contained in the SLD.

#### 2.2.4 Withdrawal criteria

(1) Voluntary withdrawal by the participant: a) Participants have the right to withdraw from the study at any stage for any reason; b) If a participant does not formally declare withdrawal but discontinues medication intake and participation in related examinations, or cannot be contacted during follow-up, they will be considered as having substantively withdrawn from the trial.
(2) Withdrawal decided by the researchers: a) Occurrence of serious adverse events (AEs) or significant organ function abnormalities during the study that could pose a risk if the participant continues in the trial; b) Poor medication compliance by the participant, taking less than 80% or more than 120% of the prescribed dose of SLD; c) Violation of the protocol by the participant, such as using other interventions that could interfere with the assessment of efficacy or safety, like amifostine, recombinant human interleukin-7 (IL-7), or thymosin α1.

### 2.3 Interventions

All enrolled patients will undergo CCRT. Radiation therapy will be delivered using intensity-modulated radiation therapy. The protocol involves conventional dose fractionation, targeting a total dose of 60-70 Gy over 30-35 fractions spanning 6-7 weeks. The radiation therapy approach will align with methodologies detailed in prior studies (9). Concurrent chemotherapy will include platinum-based regimens (cisplatin at 25 mg/m^2 on days 1-3 or carboplatin with an AUC of 5 on day 1), supplemented with other agents. For squamous cell carcinoma, pemetrexed (500 mg/m^2 on day 1) is the usual choice, while for non-squamous carcinoma, either docetaxel (135 mg/m^2) or albumin-bound paclitaxel (260 mg/m^2 on day 1) is preferred. Three chemotherapy cycles are planned, coinciding with the 1st, 4th, and 7th weeks of the radiation therapy.

In addition to CCRT, patients in the experimental group will receive SLD treatment from the first day of CCRT until the end of the 12th week. SLD is to be taken thrice daily, one dose at a time, with warm water post meals. SLD is produced into granules by Sichuan Neo-Green Pharmaceutical Technology Development Co., Ltd. (Sichuan, China), adhering to the China Production Quality Management Code. The product’s quality control procedures will be in accordance with the Pharmacopoeia of the People’s Republic of China (Chinese Pharmacopoeia Commission, 2018 version).

### 2.4 Outcomes

#### 2.4.1 Primary outcome

The primary outcome is the incidence rate of ASL, defined as the proportion of patients who experience at least one episode of grade ≥3 lymphocytopenia within 12 weeks after the start of CCRT treatment. The grading of lymphocytopenia will be based on the CECAE version 5.0 published by the National Cancer Institute (10). The grading is specifically defined as follows: Grade 0 (TLC ≥ 1.1), Grade 1 (0.8 ≤ TLC < 1.1), Grade 2 (0.5 ≤ TLC < 0.8 × 10^9/L), Grade 3 (0.2 ≤ TLC < 0.5 × 10^9/L), and Grade 4 (TLC < 0.2 × 10^9/L).

#### 2.4.2 Secondary outcomes

(1) Changes in TLC: TLC will be measured weekly from the start to the end of CCRT, and then biweekly from the end of CCRT to the 12th week post-CCRT.
(2) Changes in Peripheral Blood Lymphocyte Subsets: This includes absolute and relative counts of T lymphocytes (CD3+), helper T cells (CD3+CD4+), cytotoxic T cells (CD3+CD8+), B lymphocytes (CD19+), and NK cells (CD16 + 56+). These will be measured at baseline and once again at the 12th week post-CCRT.
(3) Changes in KPS Score: The KPS score is a standardized scale for assessing the functional status of cancer patients, with a range from 0 to 100, in increments of 10. Higher scores indicate better physical condition and quality of life. KPS will be assessed at baseline and at the 12th week post-CCRT.
(4) Short-term Efficacy of Lung Cancer Treatment: This encompasses the Objective Response Rate (ORR) and Disease Control Rate (DCR). ORR is the proportion of patients exhibiting a confirmed best response of either complete response or partial response, as per the RECIST (Response Evaluation Criteria in Solid Tumors) version 1.1 criteria (11). DCR represents the percentage of patients achieving complete response, partial response, or stable disease. Imaging examinations are scheduled 4 weeks following the completion of CCRT to evaluate the short-term efficacy of the treatment.
(5) Long-term Efficacy of Lung Cancer Treatment: This includes the rates of Progression-Free Survival (PFS) and Overall Survival (OS). PFS is defined as the duration from randomization to either the first instance of disease progression, as determined by RECIST criteria, or death. OS is the time span from randomization to death due to any cause. For effective monitoring of long-term efficacy, patients will undergo regular follow-up assessments every 3 months during the initial 3-5 years, subsequently every 6 months for the following 5 years, and then annually until disease progression or death.

#### 2.4.3 Safety outcomes

During the 12-week treatment period, AEs will be continuously monitored. AEs are defined as any adverse medical events that occur in a patient during a clinical study, which may present as symptoms, signs, diseases, or laboratory test abnormalities. Laboratory tests, including blood routine, stool routine, urine routine, liver function (i.e., γ-glutamyl transferase, alkaline phosphatase, alanine aminotransferase, aspartate aminotransferase, and total bilirubin), renal function (i.e., blood urea nitrogen and creatinine), and electrocardiogram, will be conducted weekly from the start to the end of CCRT, and biweekly thereafter until the 12th week. Any occurrence of AEs will prompt corresponding treatment measures for the patient and will be meticulously recorded in the case report forms, detailing the time of occurrence, clinical manifestations, duration, severity, frequency, treatment measures, outcomes, and the causal relationship with the trial drug SLD. The severity of AEs will be assessed using the CTCAE version 5.0 (10). Any serious AEs will be reported to the principal investigator and the ethics committee within 24 hours of occurrence. Adverse reactions are defined as AEs related to the trial drug SLD treatment. The causality between SLD and AEs will be assessed using the World Health Organization-Uppsala Monitoring Centre system (12), and AEs assessed as “definitely related,” “probably related,” or “possibly related” will be considered adverse reactions.

#### 2.4.4 Mechanism outcomes

To understand the mechanism of action by which SLD prevents ASL, this study will collect fecal and blood samples from participants at baseline and at the end of the 12-week treatment period. The aim is to analyze the gut microbiota in fecal samples through 16S rRNA gene sequencing and to conduct metabolomic analysis of gut microbiota/blood samples using gas chromatography-time of flight mass spectrometry technology. In addition, a series of bioinformatics methods will be used for in-depth analysis of the obtained data. This includes, but is not limited to, α/β diversity analysis of gut microbiota, fecal/blood metabolites, Principal Component Analysis (PCA), Principal Coordinates Analysis (PCoA), Non-metric Multidimensional Scaling (NMDS), Permutational Multivariate Analysis of Variance (PERMANOVA), and Linear Discriminant Analysis Effect Size (LEfSe).

### 2.5 Participant timeline

**Figure 2:**
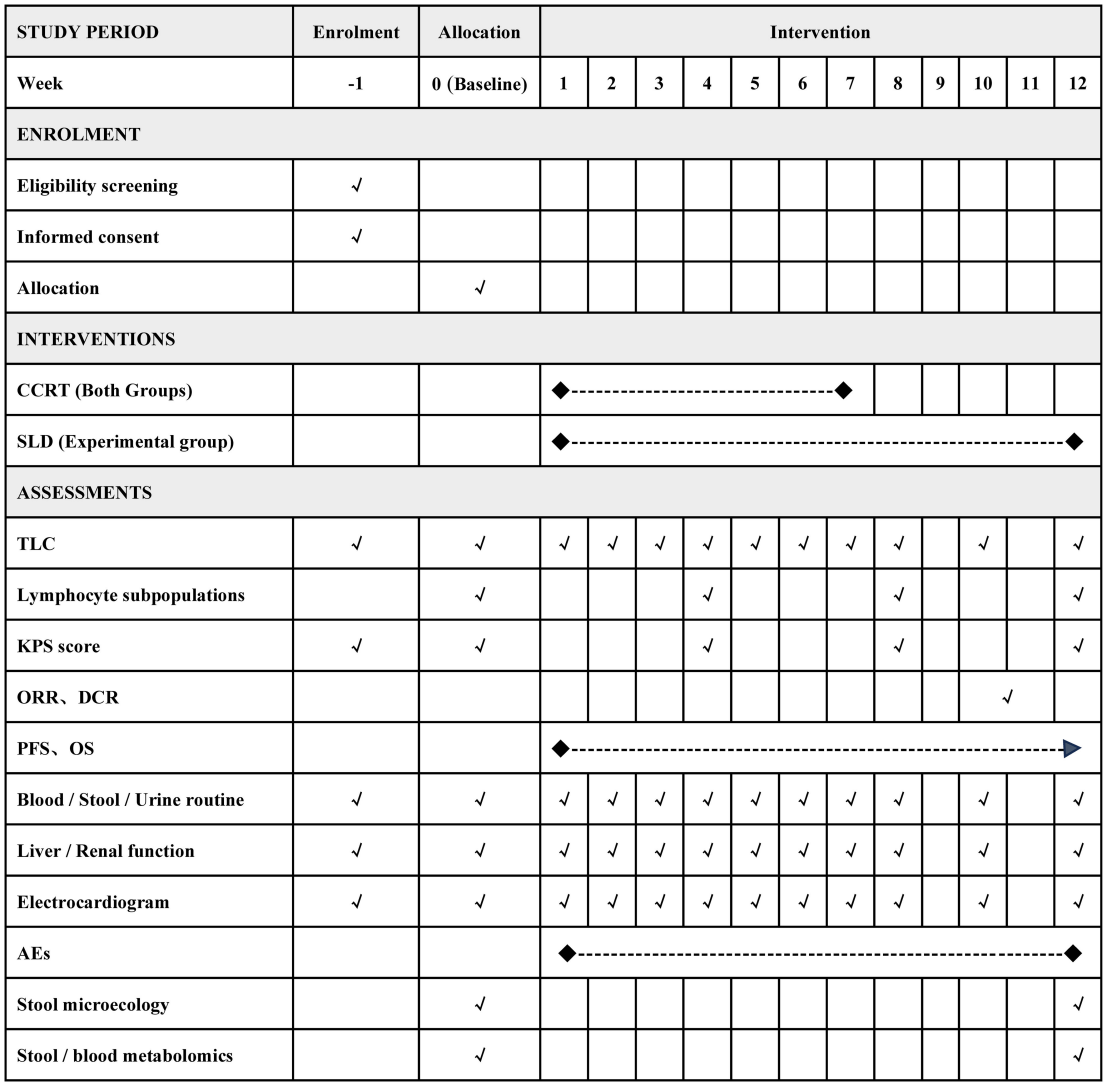
Detailed timeline of participant enrollment, intervention strategies, and assessment points. CCRT, concurrent chemoradiotherapy; SLD, shenglin decoction; TLC, total lymphocyte count; ORR, objective response rate; DCR, disease control rate; PFS, progression free survival; OS, overall survival; AEs, adverse events.

Eligible participants will undergo a 1-week pre-intervention screening followed by a 12-week treatment period. The timeframe for data collection or assessments is depicted in **Figure 2**.

### 2.6 Sample size

The primary outcome in this study is the incidence of ASL. Based on preliminary retrospective study findings, the anticipated incidence rates of ASL in the experimental and control groups are projected at 60.7% and 86.5%, respectively. Using PASS software (version 15), it is calculated that each group requires a minimum of 70 participants (bilateral α = 0.05, 1-β = 0.90, 1:1 ratio, anticipated dropout rate = 15%).

### 2.7 Randomization and blinding

The study plans to enroll 80 participants at Sichuan Cancer Hospital and 60 participants at the Hospital of Chengdu University of Traditional Chinese Medicine, stratified by research center. A third-party statistician will generate a randomization sequence for each center using block randomization methods (block sizes known only to the statistician) and keep the random codes in opaque, sealed envelopes. After obtaining informed consent, participants will receive their group assignment by sequentially opening an envelope based on their order of enrollment. This study is designed as an open-label study, meaning that both participants and doctors will be aware of the treatment allocations from the beginning of the study. To minimize bias, outcome assessors, data managers, and statisticians will remain unaware of the treatment allocations throughout the study.

### 2.8 Statistical analysis

All efficacy analyses will follow the intention-to-treat principle and include all randomized participants. For the primary efficacy outcome (incidence of ASL), prespecified sensitivity analyses will also be conducted in the assessable population (excluding participants with incomplete outcome data) and in the per-protocol population (including only participants who adhered to the treatment and did not significantly violate the study protocol). Safety analysis will include all participants who have received at least one dose of SLD and have completed at least one safety assessment post-baseline. Missing data will be handled using multiple imputation methods.

A generalized linear model with a binomial distribution will be used to estimate the difference in the incidence of ASL between groups, adjusting for multiple key variables in the model, including trial center, age, baseline TLC value, radiation dose, and chemotherapy cycles. For comparisons of changes in TLC and peripheral blood lymphocyte subsets between groups, a generalized estimating equation with repeated measures will be employed. This model will include fixed effects of trial center, group, time, group-by-time interaction, and respective baseline values. For comparisons of changes in KPS score between groups, depending on the normality of the data, either independent sample t-tests or Mann–Whitney U tests will be used. For PFS and OS, Kaplan-Meier survival curves will be presented, and hazard ratios (HR) will be calculated using a stratified Cox proportional hazards regression model that is stratified by both trial center and the use of ICI maintenance therapy. The incidence of adverse events between groups will be assessed using chi-square tests or Fisher’s exact tests.

Data analysis and visualization will be performed using SPSS (version 26.0) and R (version 4.2.1) software. All hypothesis tests will be two-sided, and a P-value of less than 0.05 will be considered statistically significant. As for secondary outcomes, no adjustments for multiple comparisons will be made, and the results should be interpreted as exploratory.

### 2.9 Quality control and data monitoring

The trial will be conducted in accordance with the ethical principles of the Declaration of Helsinki (13), the Good Clinical Practice guidelines of the International Council for Harmonization (14), and the applicable regulatory requirements of China. To ensure the quality of the trial, researchers at each participating center will receive professional training to ensure their comprehensive understanding of the entire research protocol and operational procedures. The training will cover key aspects of the trial, such as guiding participants on the correct use of SLD, filling out Clinical Research Form(CRF), reporting AEs, and recording participant dropout.

All collected data will be recorded in standardized paper-based CRF. Assessment forms, laboratory test reports, and other relevant records will be marked with codes and names. After completing the paper CRF, two research assistants at each research center will enter the collected information into the Chinese Clinical Trial Management Public Platform to ensure unified management of the information. This process will involve double data entry; in case of inconsistencies in the entered data, data verification and adjustments will be made to resolve any data discrepancies and ensure the integrity and accuracy of the data. After the completion of the study, the original paper-based CRF will be stored at the research centers for five years.

### 2.10 Trial status

As of December 20, 2023, 27 participants have been enrolled in the study. The recruitment of participants is currently ongoing.

## 3 Discussion

ASL is now widely recognized as negatively impacting the prognosis of NSCLC patients (6). A retrospective study involving 604 stage III NSCLC patients receiving CCRT found a significant association of ASL with reduced PFS (HR = 1.4, *P* = 0.02) and OS (HR = 1.5, *P* = 0.01) (4). Another retrospective analysis of 309 NSCLC patients treated with CCRT, either with or without durvalumab immunotherapy maintenance, identified prognostic factors (15). The results indicated that the occurrence of ASL was significantly associated with a shortened median OS in both treatment groups (CCRT only: median OS of 18.1 months for patients with ASL versus 45.8 months for those without ASL; CCRT plus durvalumab: 24.6 months versus not reached; *P* < 0.05 for both). In patients without ASL, the combination with durvalumab significantly prolonged median OS compared to CCRT only (45.8 months *vs*. not reached, *P* < 0.05), but this was not the case for patients who had experienced ASL (18.1 months *vs*. 24.6 months, *P* > 0.05), suggesting that the occurrence of ASL diminishes the effectiveness of maintenance immunotherapy. Another study analyzed the impact of recovery of absolute lymphocyte counts to >500/μL at 3 months post-CCRT on the prognosis of stage III NSCLC patients undergoing maintenance immunotherapy (7). It was found that lymphocyte recovery was identified as a positive prognostic factor for both PFS (HR = 0.35, *P* = 0.034) and OS (HR = 0.24, *P* = 0.007). Therefore, preventing the occurrence of ASL and facilitating its early recovery might be an important direction for improving patient outcomes in the future.

Current strategies for preventing ASL in NSCLC primarily focus on optimizing radiotherapy plans. These strategies include (1): reducing the number of radiotherapy sessions, such as employing hypofractionated radiotherapy (8) (2); minimizing radiation exposure to the immune system, particularly targeting bone marrow and spleen (16, 17) (3); adopting advanced radiotherapy technologies like proton therapy (18). Furthermore, several pharmacological agents have demonstrated potential in mitigating ASL. Amifostine, a cytoprotective agent, can prevent radiation-induced apoptosis (19), with clinical studies indicating its efficacy in reducing lymphocyte damage and consequently ASL incidence (20, 21). However, the broader clinical application of amifostine is limited due to its adverse effects, including dizziness, nausea, vomiting, fatigue, and hypotension (22). IL-7, a vital growth factor for T lymphocytes, plays a significant role in their proliferation, differentiation, and metabolism (23). Research in mouse models of intestinal and lung cancer suggests that IL-7 enhances CD8+ T cell infiltration and suppresses myeloid-derived suppressor cells, boosting the efficacy of chemotherapy and immunotherapy (24). TJ107, a long-acting recombinant IL-7, is currently being evaluated in a phase II clinical trial for its effectiveness and safety in treating lymphopenia in glioblastoma patients post-CCRT (NCT04600817). Additionally, Thymosin α1, known to facilitate T lymphocyte proliferation and differentiation (25), has shown promise in a phase II trial with stage III NSCLC patients. Patients treated with Thymosin α1 during CCRT exhibited a significantly lower ASL incidence rate of 19.1%, compared to 62.1% in the control group, as per propensity score-matched analysis (26).

In China, Traditional Chinese Medicine (TCM) is widely used to alleviate the toxic side effects of CCRT (27). Facing considerable challenges in the clinical management of ASL, TCM’s multi-target approach emerges as a vital research direction. According to TCM theory, ASL caused by CCRT is considered a deficiency of qi and blood. Bazhen Decoction (BZD), a classic formula with a history of over 700 years known for its qi and blood nourishing properties, has been shown in multiple studies to promote the proliferation and activation of lymphocytes (28). BZD consists of eight Chinese herbs, including Sheng Shai Shen [*Panax ginseng* C.A.Mey. (Araliaceae; Ginseng radix)], Shu Di Huang [*Rehmannia glutinosa* Libosch. (Orobanchaceae; Rehmanniae radix preparata)], Bai Zhu [*Atractylodes macrocephala* Koidz. (Asteraceae; Atractylodis macrocephalae rhizoma)], Fu Ling [*Poria cocos* (Schw.) Wolf. (Polyporaceae; Poria)], Dang Gui [*Angelica sinensis* (Oliv.) Diels. (Apiaceae; Angelicae sinensis radix)], Chuan Xiong [*Ligusticum chuanxiong* Hort. (Apiaceae; Chuanxiong rhizoma)], Bai Shao [*Paeonia lactiflora* Pall. (Paeoniaceae; Paeoniae radix alba)], and Gan Cao [*Glycyrrhiza uralensis* Fisch. (Fabaceae; Glycyrrhizae radix)].

Recent research further suggests that combining BZD with heat-clearing or blood-activating Chinese herbs during CCRT can enhance the effect of BZD on increasing TLC and shows potential in prolonging the survival of cancer patients (29, 30). Based on TCM theory and clinical evidence, we meticulously developed SLD, specifically for preventing ASL in stage III NSCLC patients undergoing CCRT. SLD is an improved version of BZD, with the addition of Huang Qi [*Astragalus membranaceus* (Fisch.) Bunge (Fabaceae; Astragali radix)] for its qi and blood nourishing effects, heat-clearing herbs such as Jin Yin Hua [*Lonicera japonica* Thunb. (Caprifoliaceae; Lonicerae flos)], Lian Qiao [*Forsythia suspensa* (Thunb.) Vahl (Oleaceae; Forsythiae fructus)], Mai Dong [*Ophiopogon japonicus* (Thunb.) Ker Gawl. (Asparagaceae; Ophiopogonis radix)], Nan Sha Shen [*Adenophora stricta* Miq. (Campanulaceae; Adenophorae radix)], and blood-activating herbs such as Dan Shen [*Salvia miltiorrhiza* Bunge (Lamiaceae; Salviae miltiorrhizae radix et rhizoma)], San Qi [*Panax notoginseng* (Burk.) F.H.Chen (Araliaceae; Notoginseng radix et rhizoma)].

This study is structured as a prospective, dual-center, randomized controlled trial, adhering to the CONSORT extension guidelines for Chinese herbal compound formulas (31). To delve deeper into the action mechanism, we will conduct 16S rRNA gene sequencing of participants’ gut microbiota and perform metabolomic analyses of fecal and blood samples. This holistic approach enables us to comprehensively assess SLD’s efficacy, safety, and mechanistic pathways, aiming to establish a robust evidence base. However, this study has limitations. Its open-label design may introduce bias, although primary outcomes are assessed using objective measures to minimize this. Additionally, being a dual-center study limited to China, the applicability of our findings to other populations or contexts may be constrained.

## 4 Ethics and dissemination

This study protocol has been approved by the ethics committees of both implementing institutions (approval numbers: 2023KL-058-01 from Hospital of Chengdu University of Traditional Chinese Medicine; SCCHEC-02-2023-074 from Sichuan Cancer Hospital) and is registered in the Chinese Clinical Trial Registry (registration number: ChiCTR2300071788). In accordance with the Declaration of Helsinki and local standard operating procedures/regulations, informed consent will be secured prior to initiating any procedures related to the study, including the screening process. The results of the study will be disseminated in a peer-reviewed journal following the completion of the study.

## Ethics statement

The studies involving humans were approved by the Hospital of Chengdu University of Traditional Chinese Medicine and the Sichuan Cancer Hospital. The studies were conducted in accordance with the local legislation and institutional requirements. Written informed consent for participation was obtained from the participants of the study.

## Author contributions

JD: Conceptualization, Data curation, Formal analysis, Methodology, Project administration, Supervision, Writing – original draft, Writing – review & editing. CG: Conceptualization, Project administration, Supervision, Writing – original draft, Writing – review & editing. QiX: Conceptualization, Data curation, Methodology, Project administration, Writing – original draft, Writing – review & editing. BX: Data curation, Formal analysis, Investigation, Methodology, Writing – review & editing. HL: Data curation, Formal Analysis, Investigation, Methodology, Writing – review & editing. ZW: Data curation, Formal analysis, Investigation, Visualization, Writing – review & editing. QX: Data curation, Investigation, Software, Visualization, Writing – review & editing. PG: Methodology, Writing – review & editing, Data curation. QL: Methodology, Software, Visualization, Writing – review & editing. BL: Data curation, Investigation, Writing – review & editing. YW: Investigation, Methodology, Writing – review & editing. BgL: Conceptualization, Formal analysis, Project administration, Supervision, Writing – review & editing. KX: Conceptualization, Formal analysis, Funding acquisition, Investigation, Project administration, Resources, Supervision, Writing – review & editing.
